# Sri Lankan School Student and Teacher Perspectives of Adolescent Mental Health and Its Determinants: A Qualitative Exploration

**DOI:** 10.3390/healthcare14030311

**Published:** 2026-01-26

**Authors:** Chethana Mudunna, Miyuru Chandradasa, Kavidi Amanda Epasinghe, Josefine Antoniades, Medhavi Weerasinghe, Thach Tran, Sivunadipathige Sumanasiri, Jane Fisher

**Affiliations:** 1 Global and Women’s Health, Monash School of Public Health and Preventative Medicine, 553 St. Kilda Road, Melbourne, VIC 3004, Australia; j.antoniades@latrobe.edu.au (J.A.); medhavi.weerasinghe1@monash.edu (M.W.); thach.tran@monash.edu (T.T.); 2 Department of Psychiatry, Faculty of Medicine, University of Kelaniya, Thalagolla Road, P.O. Box 6, Ragama 11010, Sri Lanka; miyuruc@kln.ac.lk (M.C.); amyepa98@gmail.com (K.A.E.); susari@gmail.com (S.S.); 3 School of Humanities & Social Sciences, La Trobe University, Bundoora, VIC 3083, Australia; 4 National Ageing and Research Institute, 34-54 Poplar Road, Parkville, VIC 3052, Australia; 5 Biostatistics Unit, Faculty of Health, Deakin University, Burwood, VIC 3125, Australia

**Keywords:** mental health, adolescent, student, teacher, school, Sri Lanka

## Abstract

**Highlights:**

**What are the main findings?**

- Sri Lankan adolescents’ and teachers’ understanding of mental health is predominantly rooted in Buddhist perspectives.
- School environment plays a central role in exacerbating risk factors to poor mental health in adolescents.
- Sri Lankan adolescents have more knowledge of informal support sources rather than formal support for mental health-related concerns.

**What are the implications of the main findings?**

- There is an opportunity for mental health promotion in Sri Lanka to leverage culturally contextualised language and frameworks.
- Schools are a key place to promote mental health to adolescents by integrating mental health programmes into the school curriculum and mental health promotion into routine educational practice.

**Abstract:**

**Background/Objectives**: Across geographical and cultural contexts, how individuals identify, communicate and help-seek for distress is often shaped by how mental health itself is understood. Insight into how adolescents and adults in their routine environment, such as teachers, understand mental health is crucial for developing context-specific mental health promotion strategies to young people. Sri Lanka, a country that navigates the dual legacies of pre-and-post-colonial mental health frameworks, has this need. The aim was to explore Sri Lankan school-going adolescents’ and their teachers’ perspectives of mental health and its determinants. **Methods**: Semi-structured interviews were conducted with 28 school-going adolescents in grades 10–12/13 and 14 of their school teachers, from seven secondary schools in Gampaha District, Sri Lanka. Interviews were transcribed, translated, coded inductively and analysed thematically. **Results**: All participants drew on culturally meaningful language that is rooted in Buddhist perspectives to conceptualise mental health. Causes and risk factors of poor mental health were attributed to individual, immediate environmental and structural factors. School environment played a central role in exacerbating other risk factors. Adolescents exhibited more knowledge of informal care avenues for mental health-related concerns. **Conclusions**: Findings highlight several implications including opportunities to leverage culturally contextualised language/frameworks when promoting mental health to Sri Lankan adolescents, diversifying mental health research and initiating school-based mental health programmes that integrate mental health promotion into routine educational practice to transform learning institutions across Sri Lanka to become mental health-promoting schools.

## 1. Introduction

Current psychiatric nosology is primarily based on identifying patterns of distress, which are then categorised into standardised diagnoses based on operational criteria [1]. Yet, across geographical and cultural contexts, the prevalence, symptoms and outcomes of psychiatric illnesses vary [2]. These differences reflect diverse conceptualisations of mental health, which shape how individuals identify, communicate and seek help for distress, and how mental health problems are diagnosed, treated and researched [2,3]. Identifying context-specific understandings of mental health is therefore important to building a foundation for local targeted responses for mental health problems.

Adolescence is a critical period of human development in which to examine understandings of mental health, because a cascade of developmental changes occurs between the ages of 10 and 19 years [4,5]. These include rapid physical, emotional and social growth, as well as the establishment of life-long patterns of behaviour related to diet, exercise and sexual activity [4,5,6]. Adolescents are particularly vulnerable to experiencing challenges to their mental wellbeing to varying degrees during this unique phase of development [4,6]. Adding to this, approximately 50% of mental health problems manifest by the age of 15 [6]. Poor mental health during adolescence is further associated with poor outcomes in educational attainment and physical health in adulthood [4]. Insight into how adolescents themselves understand mental health can inform interventions that strengthen knowledge, attitudes and behaviours needed for adolescents to manage thoughts and emotions effectively to build a strong, positive sense of identity and healthy social and family relationships [7].

Schools are key settings for such interventions. Teachers, who spend the most time with school-going adolescents, play a vital role in shaping the school milieu, classroom climate, social and emotional skill development, promotion of good mental wellbeing and setting help-seeking norms [8,9]. Understanding teachers’ perspectives on mental health is therefore essential to fostering a supportive and positive school environment and integrating mental health promotion into routine educational practice.

In low-and-middle-income countries (LMICs), there are low rates of mental health problem recognition, large treatment gaps and several structural barriers to help-seeking [4,10,11]. A recent systematic review found that children and adolescents in LMICs have low levels of recognition and knowledge about mental health, and experience stigma and low confidence in help-seeking from formal care avenues [10]. Qualitative studies conducted in LMICs such as Indonesia found that adolescents often equate poor mental health with serious or severe mental illness manifesting as emotional, behavioural and physical disturbance [12]. Identifying how mental health and its determinants are understood by young people and influential adults around them is an important first step towards narrowing global mental health inequities and developing context-specific interventions for adolescents living in LMICs.

Sri Lanka is an LMIC in South Asia with a population of 22 million people, where one-fifth are adolescents [13]. Mental health in Sri Lanka has been shaped by a complex interplay of spiritual, sociocultural and political forces over time. Buddhist and Hindu beliefs, alongside Indigenous Ayurvedic traditions, formed the foundations of pre-colonial conceptualisations of mental health in Sri Lanka. Mental suffering and distress were viewed through a spiritual lens, where it was understood as a natural part of human existence [14]. Particularly in Buddhism, the dominant religion in Sri Lanka, the core philosophies that shape interpretations of emotions, wellbeing and suffering, such as the philosophy of *Dukkha*, suggests that suffering is a part of life [15]. The regulation of emotions through mindfulness and harmony of inter- and intra-personal relationships is also described in Buddhist frameworks around mental health [15]. During British colonial rule, mental health was reframed with a pathological framework, where “non-normative” thoughts, behaviours and feelings were treated as disorders and a distinction was made between ‘disorder’ and ‘normalcy’ [14,16]. Despite evidence of high prevalence of mental health problems and the influence of social determinants on adolescent mental health [9,17], data on how Sri Lankan adolescents and teachers currently understand mental health itself remain scarce. Sri Lanka has a need for context-specific understandings of mental health, which can help enhance mental health promotion targeted towards young people.

The overall aim of this study was descriptive. It was to describe Sri Lankan school-going adolescents’ and their teachers’ perspectives of mental health and its determinants. Guided by this aim, this study addresses the following research question: What are Sri Lankan adolescents’ and teachers’ perspectives of mental health, its determinants and available care avenues? The specific research objectives looked to describe (i) knowledge and attitudes about mental health; (ii) causes and risk/associated factors of poor mental health outcomes; and (iii) knowledge of care avenues.

## 2. Materials and Methods

### 2.1. Study Design

This study is the qualitative arm of an explanatory, sequential, mixed-methods study. A systematic review, a policy review and a quantitative study using a cross-sectional survey preceded this qualitative study. While the quantitative component provided an understanding of adolescent mental health in Sri Lanka, semi-structured interviews were used in this study to further refine and explain the findings from the quantitative component [18]. Interviews were conducted with school-going Sri Lankan adolescents and their teachers. The interviews took place between July and September 2023.

### 2.2. Study Setting

The interviews were conducted in the administrative division of Gampaha, in the Gampaha zone of Gampaha District, in Western Province, Sri Lanka. It is the second most populated district in the country, with a reported population in 2021 of 2.4 million people [19]. Gampaha District has a multi-ethnic and multi-religious population. Most people are Sinhalese (90.5%) and Buddhist (71.3%). Sri Lankan Moors make up 4.2% of the population in the district and Sri Lankan Tamils make up 3.9% of the population [19]. There are around 350,000 adolescents residing in Gampaha District [19], making up around 15% of the total population of Gampaha.

### 2.3. Study Sites

The study sites were seven secondary schools in Gampaha District. A stratified random sampling technique was used to select schools for the multiple methods study. Government schools in Gampaha Division were stratified into urban, semi-urban and rural streams. Then, four urban, one semi-urban and two rural schools were randomly selected by an independent statistician who then selected two classes from each of grades 10, 11, and 12/13 randomly. Where schools had both Sinhala- and English-medium streams of education, one Sinhala- and one English-medium class were selected from each year level. Where two streams of education did not exist, two classes were randomly selected from each of the above-mentioned year levels.

### 2.4. Participants and Recruitment

Adolescents from the selected classes who had completed the quantitative component of the multiple methods study were invited to volunteer for interviews. The opportunity to participate was given to any teachers interested. To manage feasibility and time-constraints, up to four adolescents and two teachers from each school were able to volunteer to participate in the study.

#### Inclusion and Exclusion Criteria

Adolescents in grades 10, 11, 12/13, attending seven selected Sinhala and English medium schools in Gampaha District, Sri Lanka, who had also completed the quantitative component of the multiple methods study, were included.

Teachers at seven selected Sinhala and English medium schools in Gampaha District, Sri Lanka were included.

Students with an inability to speak, even with support due to physical or psychological disability, and students and teachers absent on the day of interview administration were excluded.

### 2.5. Procedure

First, the researchers requested permission from school principals to conduct the multiple methods study in each selected school. Once permission was obtained, principals elected a project coordinating teacher at each of the schools to manage the multiple methods study and notify all teachers and parents.

Next, several community meetings were held via Zoom with school staff, the research team and parents to inform them about the aims and objectives of the multiple methods study. Following this, information sheets, consent forms and objection forms were sent to parents of adolescents under the age of 18 to obtain consent to participate in the multiple methods study. All adolescents who returned parental signed consent forms and met eligibility criteria were able to volunteer to participate in interviews.

Prior to the interviews, signed assent was obtained from all adolescent volunteers under 18. Signed consent was obtained from all adolescent volunteers over 18 and from all volunteer teachers.

Participants were offered the option for interviews to be conducted in Sinhala or English. All participants selected for interviews to be conducted in Sinhala.

To ensure that participants understood their rights as interview participants, the researcher provided a verbal explanation about the procedure of the interviews in their language of choice once more. This included notification of the following: (1) interviews are being recorded; (2) signing consent and assent forms informs consent to be recorded; (3) the interviews are voluntary; (4) adolescent participants were notified that the interviews were not part of their standard syllabus; (5) all participants were clearly informed that they could withdraw consent and not complete the interview at any point.

Interviews were conducted by a local Research Officer, who is a member of the research team, and has decades of expertise in conducting fieldwork in adolescent mental health across LMICs. Interviews were also conducted by the primary investigator, a bilingual speaker, who also has experience conducting semi-structured interviews.

### 2.6. Instruments

An interview guide with semi-structured open-ended interview questions was developed in advance (Table 1). The researchers identified evidence gaps, generated interview questions and ensured content validity and sensitivity of questions based on our systematic review Mudunna et al. 2025 [9] of adolescent mental health in South Asia. A similar study on adolescent mental wellbeing conducted by Willenberg et al. 2020 [12] in Indonesia was also used as a guide to generate interview questions. Both studies are published in peer-reviewed journals and publicly accessible.

The interview questions were translated to Sinhala using a forward–backward translation method. They were forward translated by one clinician and the primary investigator, who are bilingual speakers, separately. Next, the translations were compared and backward-translated, and a final version of the interview questions was produced. The accuracy of translations was reviewed by a member of the research team, who is a bilingual speaker.

### 2.7. Data Management and Analysis

Data were first transcribed verbatim to Sinhala and then translated to English by a local researcher, who has experience in transcription and translation. Next, data were analysed by two members of the research team using inductive thematic analysis, where the themes identified were linked to the data and there was no pre-existing coding framework [20]. This process included following the six steps of thematic analysis set forth by Braun & Clarke, 2006 [20]: familiarising oneself with the data, searching for themes, reviewing themes, defining themes, naming themes and producing a final analysis. An excel-matrix-based framework was developed through this initial coding and analysis process and researchers identified quotes that broadly aligned with the developed coding framework (see Supplementary Materials) [21]. This form of excel-based framework was developed because the research team was based internationally and not all members of the research team had access to qualitative data analysis software such as NVivo. The analysis was informed by a pragmatic interpretivist epistemological positioning, recognising that understandings of mental health are socially and culturally influenced and maybe best explored through participants’ accounts.

The primary coder initially conducted line-by-line coding of nine transcripts (21%) to inductively develop a preliminary coding framework. This framework was iteratively refined through discussion with a second researcher, with emerging themes and subthemes generated to reflect the meanings interpreted from the data.

To enhance credibility and intercoder reliability, a third researcher from the team independently applied the revised framework to a subset of transcripts. The primary coder and the third researcher met to review and reconcile coding discrepancies through discussion, updating the framework as needed. Coding by the primary and third researcher continued independently until an 80% agreement threshold was reached, at which point the coding framework was finalised. To confirm consistency and dependability, three transcripts (7%) were jointly coded using the final framework.

Once the excel-matrix-based coding framework was finalised by the research team, the primary coder coded the remaining transcripts on NVivo [22] using the developed framework. Illustrative quotes obtained through coding were further analysed and interpretated.

### 2.8. Reflexivity

The research team engaged in reflexive practices throughout data collection and analysis. The data collection team maintained a reflective journal to record observations, methodological decisions and reflections on how their positionality might influence interactions with the participants or the interpretation of the data. Data collectors all shared linguistic and cultural backgrounds with participants, which supported nuanced understanding of how participants understood mental health. To mitigate potential influence of shared assumptions and biases, the research team met regularly to discuss findings and interpretations, so it was ensured that interpretations were all grounded in the participants’ beliefs and narratives.

### 2.9. Ethics Approval

Ethics approval was obtained for the overall multiple methods study, which included this qualitative study. First, ethics approval was obtained from the University of Kelaniya, Faculty of Medicine Ethics Review Committee (ID: P/124/09/2022). This was followed by project registration with the Monash University Human Research Ethics Committee (ID: 37225). Next, fieldwork approval was obtained from the Department of Education, Western Province, Sri Lanka (ID: WP/ED/DEV/13/V); the Line Ministry of Education (ID: ED/03/56/02); and finally, the Zonal Education Office of Gampaha Zone, Gampaha District, Sri Lanka.

## 3. Results

The study sample comprised 28 adolescents and 14 teachers. All volunteers agreed to be interviewed. All participants’ demographic characteristics are presented in Table 2.

Three overarching themes were identified. The first theme, knowledge and attitudes towards mental health, captured participants’ general knowledge, conceptual understanding of ‘good’ and ‘poor’ mental health, and their attitudes towards mental health and people living with mental health problems. The second, beliefs of causes and risk factors, reflected participants’ perceptions of the various influences within both their immediate and broader environments that impact the mental wellbeing of Sri Lankan adolescents. The third theme included knowledge of care avenues, which encompassed their awareness of both formal and informal support pathways that may be available for mental health concerns.

### 3.1. Knowledge and Attitudes Towards Mental Health

Most participants described good mental health in terms such as “stable”, “positive”, “free” and “balance”, which are grounded in Buddhist ideals of mindfulness. For example, a rural-dwelling female adolescent suggested that people with good mental health are “*maintaining a positive and stable mental state. They behave well in society and communicate effectively with others*”. Additionally, good mental health was linked to having a ‘balanced’ mind:

“*It is clear that a person with good mental health has a highly balanced mind. When you have a problem, you can recognise that if it is a good mental health condition, can think and decide something without getting worried. Has a balanced mind*”

—Semi-urban-dwelling, female, teacher

Extending on this, an urban-dwelling male and a female adolescent also mentioned that good mental health is “*being in a positive mental state, free from issues”*, and *“mental freedom in a certain environment*”.

Poor mental health was commonly seen as inability to cope with daily stressors or challenges. For example, an urban-dwelling female adolescent suggested that people with poor mental health are “*Susceptible to stress, Unable to face challenges*”. Further, a rural-dwelling female adolescent stated that the words ‘poor mental health’ “*makes me think of people struggling with stress. They often make poor decisions and may bother others*”. It was also suggested that people with poor mental health tend to have a negative mindset:

“*[They] always have a negative outlook. They tend to focus on the negative aspects of situations and often try to avoid or run away from problems*”

—Semi-urban-dwelling, female, adolescent

Across participants, stigmatising attitudes towards people with mental health problems were identified. These attitudes were predominantly in the form of public stigma, where participants believed that the community had negative attitudes, beliefs and stereotypes towards people with mental health problems. Participants identified discriminatory language that may be used by others to refer to people with mental health problems:

“*They may be referred to as a ‘madman’ in some instances. They might be labelled as a ‘fool’*”

—An urban-dwelling, male, adolescent and a rural-dwelling, female, adolescent

“*They often use words like ‘mongol.’ ‘Crazy’ is the term used*”

—Urban-dwelling, female, adolescent

Additionally, participants highlighted that these stigmatising attitudes can contribute to social exclusion of persons living with mental health problems:

“*They are often ostracised from society. People often make hurtful comments to them*”

—Rural-dwelling, female, adolescent

### 3.2. Beliefs of Causes and Risk Factors

Participants identified several causes and risk factors of poor mental health in Sri Lankan adolescents across multiple layers of an adolescent’s immediate and surrounding environment.

#### 3.2.1. Individual Factors

Most participants linked higher age to increased individuality, more responsibilities and more life challenges, thereby suggesting that older adolescents were at risk of facing poorer mental health outcomes:

“*When they become teenagers and gain independence, problems increase. They start to make decisions on their own, and sometimes relationship issues lead to mental health challenges. They also take on responsibilities in their families, which can bring more pressure*”

—Urban-dwelling, female, adolescent

Adding to this, participants also suggested that females were more likely to have greater responsibilities and societal pressures, and therefore face a greater risk of poor mental health outcomes:

“*A woman has more stress than a man. Because she may have a family, she can be a wife. She can be a mother with the life she leads. There are those mothers who don’t dress for work. Not eating or drinking properly. There is no time to think about her first. I think it’s a little too much [stress] for that group*”

—Urban-dwelling, male, adolescent

#### 3.2.2. Immediate Environment

In the immediate environment of a Sri Lankan adolescent, participants identified risk factors in the home and school environments. Further, causes of poor mental health were linked to relationship problems and violent victimisation.

In the home environment, poor mental health outcomes among adolescents were mostly linked to instability among caregivers, predominantly resulting from caregiver separation: “*There are parental separations. Separation of parents is not divorce but living separately. There are the most difficult situations. Migrations, separation from parents, separation due to financial problems*”—Semi-urban-dwelling, female, teacher.

Extending on this, some participants highlighted that neglect by caregivers can result in adolescents engaging in risk-taking behaviours: “*One issue is that many children are neglected by their parents due to the parents’ own problems. This neglect can lead children to turn to unhealthy coping mechanisms like substance abuse to alleviate their stress*”—Semi-urban-dwelling, female, teacher.

The school environment was identified by most participants as a key driver of poor mental health outcomes. Participants suggested that academic pressure and expectations originate at home, primarily due to parents spending significant amounts of money on an adolescents’ education:

“*Tuition is paid from home. Because of that, I see some people coming to tuition from home rather than school. Because they pay money for tuition.*”

—Urban-dwelling, male, adolescent

As a result, adolescents then place pressure and expectations on themselves, as described by one participant: “*My brother, who is in fourth grade, faces a lot of pressure… often there’s pressure created either at home or by the students themselves. If they fail a class, they feel ashamed. Because of these kinds of situations, they often put a lot of pressure on themselves*”—Urban-dwelling, female, adolescent.

Further, participants suggested that this pressure then produces academic competition: “*There is significant competition among children, often exacerbated by the pressure parents place on them. While some children manage to maintain good mental health, the overall environment tends to create excessive stress and pressure for many*” (Urban-dwelling, male, adolescent). This competition in some instances, results in bullying among peers: “*If someone is performing well, others try to bring them down, often spreading false rumours.*” (Urban-dwelling, male, adolescent).

Adding to this, risk factors in the school environment are further exacerbated when teachers remove extra-curricular activities for students in order to accommodate academic studies, thereby removing a key stress relief outlet for adolescents. The compounding effects of all these factors create risk for poor mental health outcomes in the school environment:

“*Now they have stopped [sports] because the academic pressure is too high. If you play an extra sport, the school monitors your attendance closely, and if you miss two or three periods, you’ll be reprimanded the next morning*”

—Urban-dwelling, male, adolescent

Participants further identified peer and romantic relationships as causes for stress among adolescents. For example, an urban-dwelling female adolescent stated that “*Most of the time [adolescents] get into relationships as soon as they come of age*”. Meanwhile, an urban-dwelling female teacher alludes to peer pressure being a cause of stress for Sri Lankan adolescents: “*I think the biggest reason is the influence of others because [adolescents] think that their happiness depends on others*”.

Violent victimisation experienced in the school environment was also given as a cause of stress or distress experienced by adolescents. Mostly urban-dwelling male adolescents noted physical violence from teachers:

“*Children are beaten and scolded. There are usually some teachers that beat with hands and feet. That means they beat the children, even with their legs…Physical abuse.*”

Although one urban-dwelling female adolescent indicated experience of physical abuse from teachers regardless of being male or female: “*My class teacher tends to get angry quickly and sometimes hits the students. He doesn’t differentiate between girls and boys; if they make a mistake, he’ll hit them*”.

#### 3.2.3. Structural Factors

Most participants suggested structural causes and risk factors for poor mental health that were often beyond the individual control of Sri Lankan adolescents. The most noted factor was the rigorous, traditional education system in Sri Lanka, which participants believed creates academic pressure from a young age:

“*We talked about the mental pressure beyond those 15 or 16, but that mental pressure was created at that time. In childhood, children at one and two have nothing, and after that, at the age of six, a child becomes like a strange machine. Like a robot*”

—Rural-dwelling, female, teacher

Additionally, participants suggested that a majority of adolescents often take up tuition classes, which provides them with supplementary educational support outside of the traditional school settings in order to be able to compete in the demanding academic environment in Sri Lanka. However, this additional workload then creates an even greater amount of pressure for young people:

“*School doesn’t give an unbearable workload. However, with tuition classes, it’s hard to balance schoolwork. I also have a transportation issue…which makes my days long. Morning commute and late return with additional tuition classes make time management difficult. There’s no overwhelming workload at school, but balancing everything is the challenge*”

—Urban-dwelling female adolescent

Participants across rural and urban demographics described experiences of hardship as a result of the compounding effects of the COVID-19 pandemic and economic crisis in Sri Lanka. Some described its effects on the education of adolescents: “Since both my twin and I are doing A/Ls [Advanced Level Examinations], the expenses are high. My parents give us all they can, but with the rising costs, it’s hard. Even school-related expenses, like class fees, have increased” (urban-dwelling, female, adolescent). Expanding on this, others highlight the lack of access to basic resources and necessities for adolescents contributing to the mental vulnerabilities of Sri Lankan adolescents:

“*Most of the children come to school in the midst of economic difficulties. Now, with the current economy, it is very difficult to buy even a pair of shoes for children. Even a normal canvas is 3000 [rupees] now. The children’s shoes are torn. When they go to school wearing such shoes and compare with other students the mental level of that child is really in a bad place*”

—Urban-dwelling, female, teacher

“*This means sometimes due to the situation at home these days due to the crisis, some schoolchildren are at a state where they cannot even afford a meal. Sometimes we see what students bring as lunch, it’s very hard to watch*”

—Rural-dwelling, female, teacher

### 3.3. Knowledge of Care Avenues

Several formal and informal care avenues were identified by adolescent participants. Most identified mental health professionals as a formal source of support for mental health-related concerns: “*It’s better to see a therapist or a psychologist for such issues*” (Rural-dwelling, female, adolescent). Some adolescents exhibited knowledge of a mental health hotline to call in the case of severe distress: “*That’s why there is a suicide prevention hotline*” (Urban-dwelling, male, adolescent). Additionally, most adolescents indicated comfort in speaking with their school teachers regarding any mental health-related concerns:

“*Some problems can be discussed with teachers*”

—Semi-urban-dwelling, female, adolescent

In some schools, speaking to a school counsellor was actively encouraged:

“*The counselling teacher is usually available, and students often discuss their issues with her. I haven’t needed to go myself, but I’ve noticed that the sick room is used both for health and counselling. On days when doctors visit, we’re informed in advance, and we’re encouraged to go if we have any physical or mental health concerns*”

—Urban-dwelling, female, adolescent

Adolescents indicated greater knowledge of informal care avenues for mental health-related concerns than formal care avenues. Some suggested they relieve stress through physical activity: “*Whenever I feel angry, swimming helps me release that tension, and the physical exertion also relieves my stress*” (Urban-dwelling, male, adolescent). While others preferred creative activities:

“*Musical films are especially helpful because they have a positive vibe*”

—Urban-dwelling, male, adolescent

“*I have a friend who draws when he is angry*”

—Urban-dwelling, female, adolescent

Some adolescents suggested informal support sources tied to Buddhist practices including mood regulation and breathing techniques taught in Buddhism:

“*Techniques such as finding a quiet place, sitting comfortably, and consciously focusing on breathing while studying to keep the mind focused on the task at hand*”

—Urban-dwelling, male, adolescent

Many also highlighted that talking about their stress or problems with others is very helpful. This included friends, siblings or a parent:

“*Rather than keeping something in your mind, if you can tell it to someone else, your mind will be freed*”

—Urban-dwelling, male, adolescent

“*Parents have cared for us since childhood, so opening up to them can help alleviate sadness through conversation*”

—Urban-dwelling, male, adolescent

## 4. Discussion

Three themes were identified in this study: knowledge and attitudes towards mental health, beliefs of causes and risk factors of poor mental health or mental health problems and knowledge of care avenues. From these three themes, there were four main findings from the overall study. First, Sri Lankan adolescents’ and teachers’ understandings of mental health are closely aligned with Buddhist perspectives, which emphasise balance and impermanence of suffering. Second, participants identified determinants of adolescent mental health spanning across multiple environments that directly and indirectly influence a Sri Lankan adolescent. Third, schools were suggested as key drivers of poor mental health outcomes in Sri Lankan adolescents. Finally, adolescents indicated a preference for informal rather than professional support sources.

### 4.1. Knowledge and Attitudes

Our findings on adolescents’ and teachers’ knowledge of mental health are consistent with those of Attygalle, Perera & Jayamanne 2017 [23], who investigated mental health literacy and found that Sri Lankan adolescents appear to be able to recognise symptoms of poor mental health. Although Attygalle, Perera & Jayamanne 2017 [23] did not investigate knowledge of teachers, our study findings indicate similar abilities to recognise symptoms of poor mental health among Sri Lankan school teachers as well.

Descriptors about “freedom”, “stability”, “positivity” and “balance” used by participants when referring to good mental health point to an understanding of mental health that is rooted in Buddhist perspectives, where suffering is attributed to an ‘imbalance’ [15,24,25]. Participants drew on culturally meaningful language rather than biomedical definitions of mental health. These findings align with the pre-colonial mental health frameworks in Sri Lanka, which highlight spiritual interpretations [14]. Similar findings have been observed in Asian cultures, where optimal mental wellbeing is conceptualised through Buddhist teachings, such as a ‘harmony’. In other words, a form of ‘balance’ or ‘stability’ between the mind, body and environment [15,24]. Interestingly, these culturally grounded understandings also align with the World Health Organization (WHO) definition of mental health, which takes a more holistic approach than biomedical definitions of mental health. For instance, the WHO defines mental wellbeing as not merely the absence of illness but as a state of wellbeing in which individuals realise their potential and cope with the stressors of life, work productively and contribute to their community [26]. This definition encompasses positive functioning, in addition to overall emotional, psychological and social wellbeing, similar to the descriptors used by study participants. Comparatively, biomedical definitions of mental health are characterised by viewing mental health as the absence of disorders.

Respondents appeared empathic towards people with poor mental health. They recognised that sometimes people in the community can use negative language to describe people with mental health problems and how isolating experiencing mental health problems can be. There is inconsistent evidence of the interrelationship between knowledge of mental health itself and negative attitudes towards people with mental health problems [10]. Some studies in high-income countries (HIC) highlight that various education and behaviour-oriented anti-stigma campaigns and interventions enacted over decades have shown favourable outcomes towards de-stigmatising mental health problems [27]. However, contrastingly, other studies have found that social rejection of people with poor mental health has remained, even with more knowledge of mental health [28]. Therefore, it is unclear whether more knowledge of mental health in the wider Sri Lankan society can contribute to reducing negative attitudes towards people with mental health problems.

### 4.2. Beliefs of Causes and Risk Factors

The causes and risk factors of poor mental health identified by participants in this study align with the growing body of international literature that demonstrates the influence of social determinants on mental health [29,30]. This literature highlights that a person’s mental health can be shaped by various factors in social, economic and physical environments operating at numerous life stages of an individual [30]. These can be factors that are unique to the individual such as factors in the school and home environments, similar to those identified by our study participants. It can also be wider structural factors that generate and perpetuate intergenerational cycles of disadvantage and poor mental health [29]. For instance, poverty and resource limitations such as those identified by Sri Lankan adolescents and teachers in this study.

Notably, the tendency of participants to attribute causes and risk factors to external stressors also reflects Buddhist conceptualisations of suffering. A central Buddhist philosophy, *Dukkha*, is the acknowledgement that ‘life is full of suffering’ [15,25]. Buddhist frameworks emphasise mental distress as less of an individual pathology but rather as a more situational imbalance influenced by surrounding environments and interpersonal relationships [15]. Considering that schools and institutions in Sri Lanka are guided by Buddhist principles and that Buddhist practices are taught in many schools, it is likely that Buddhist knowledge and practices have shaped our study participants’ beliefs about the causes and risk factors of poor mental health [31]. This framing may reinforce the perception that mental distress is a part of the human experience, thereby influencing help-seeking beliefs and behaviours among adolescents and teachers.

Emerging research such as Christou et al. 2025 [32] describes the influence of social affiliation and emotional sensitivity on the social and emotional wellbeing of children. For Sri Lankan adolescents, their interpretation of beliefs and causes of risk factors to poor mental health outcomes are closely tied to interpersonal relationships and the surrounding environment. For future research, these findings by Christou et al. 2025 [32] are relevant in providing a bridge between individual emotional experiences and culturally embedded beliefs about mental health.

The risk factors reported by participants in this study—age, gender, family and school environment, peer relationships, violent victimisation, resource limitation—support and expand upon the findings of our recent large-scale systematic review, Mudunna et al. (2025), which examined determinants of mental health problems among South Asian adolescents [9]. In this current study, most notably, school environment emerges as the primary contributor to poor mental health in this population. As described by this study’s participants, this is due to its central role in amplifying other risk factors. For instance, participants described academic pressure as originating at home, but it is exacerbated in a school setting due to academic competition, removal of extra-curricular activities and the nature of the schooling system itself. Furthermore, peer pressure is also played out in school contexts, and the incidents of violent victimisation were also reported in school settings. Hence, school plays a central role where multiple, intersecting risk factors are exacerbated. This finding supports WHO’s mental health-promoting schools initiative, where schools can become sites for mental health-promoting interventions [33].

Moreover, findings suggest that recent back-to-back national crises—the COVID-19 pandemic, ongoing economic crisis, and socio-political instability—have created new challenges for Sri Lankan adolescents and teachers, as well as widened existing inequities in the country [13]. For example, participants believed that the compounding effects of these crises have caused many families to experience financial hardship, including students and teachers. These findings are supported by a recent study by Senevirathne et al. 2025 [34], which highlights the association between financial hardships that prompted lifestyle modifications among Sri Lankan school teachers, and adverse mental health outcomes. In our study, overall hardship because of compounding crises in the country were reported by both urban and rural participants, indicating experience of structural determinants of mental health regardless of area of residence. Our findings align with the broader literature on structural determinants of mental health, which suggests that crises often intensify pre-existing vulnerabilities in disadvantaged populations [35].

### 4.3. Knowledge of Care Avenues

In our study, questions about the knowledge of care avenues were only asked of adolescents, a majority of whom highlighted informal care avenues such as physical and creative activities, or talking to friends and family, as appropriate ways to alleviate distress. Our findings are consistent with international research that highlights a preference for informal support for poor mental health among young people [36]. It is suggested that this knowledge stems from various factors including shared understanding, where adolescents may feel more comfortable confiding in peers that are also going through similar experiences [37]; easier access to informal support, as adolescents do not have autonomous access to professional help and require caregivers to action concerns [36]; social relationships, where relationships with family and friends play a key role in an adolescent’s quality of life [37]; and perceived stigma, where adolescents may feel stigmatising attitudes from peers for seeking professional help, thereby making informal support more appealing [38].

In addition, these preferences for informal support sources align with Sri Lankan adolescents’ broader understanding of mental health through Buddhist perspectives, where suffering is understood to be a part of life. Therefore, it is possible that rather than seeking support from clinical and more formal sources, adolescents may prefer informal support to alleviate distress.

Notably adolescents displayed positive attitudes towards seeking help from teachers. Given that schools are key facilitators of risk factors for poor mental health in Sri Lankan adolescents, it is perhaps important and more sustainable to assist school teachers to be well-informed on promoting mental health to adolescents [8,39]. Ginige et al. 2021 [40], a study which investigated the impact of a specialised training programme on the mental health literacy of Sri Lankan school teachers, reflects this potential. Findings from Ginige et al. 2021 [40] show a significant positive impact on improving teachers’ knowledge of common child and adolescent mental health problems through the programme. Adding to this, teachers themselves can experience stress and emotional burdens, which can affect how they support students. Hence, enhancing existing programmes and helping teachers to develop support skills may be key to assisting school teachers in promoting mental wellbeing to adolescents.

### 4.4. Strengths and Limitations

This study had several strengths. There was diversity among the participants, which provides rich, in-depth insights into how Sri Lankan adolescents and school teachers understand mental health. With diverse data, trends and patterns could be identified, allowing for a more nuanced understanding of the problem. The open-ended nature of the semi-structured interviews allowed for a wide range of elaborated responses. Participants were not restricted to fixed responses to questions. Further, participants were offered the option for interviews to be conducted in Sinhala or English, the two most prominent languages spoken in Gampaha District.

We nevertheless acknowledge some limitations. We acknowledge groups who could not participate in the interviews, such as Tamil speakers and people from remote settings. Further, due to the descriptive nature of this study, causal associations cannot be established. The findings reflect participants’ interpretations of mental health, perceived influences of mental health outcomes and perceptions of care avenues. The study provides contextual understandings rather than causal evidence.

### 4.5. Implications

Findings from this study can inform culturally appropriate and age-specific strategies and interventions to promote adolescent mental wellbeing in Sri Lanka and in similar LMIC settings. Several implications are noted:

1. There is an opportunity for mental health promotion in Sri Lanka to leverage culturally contextualised language and frameworks that resonate with the beliefs of adolescents. Promotion strategies can incorporate locally meaningful language such as “balance”, “freedom” and “stability” in awareness campaigns to align more with Sri Lankan adolescents’ conceptualisation of good mental health. Framing mental health in this way also shifts its understanding from an ‘avoiding illness’ approach to a more positive approach of achieving these positive states of wellbeing. This framing can also contribute to de-stigmatising mental health by framing mental health as an attainable aspect of everyday life rather than something associated with crisis or clinical settings.
2. More public health research is needed to understand how mental health knowledge influences stigmatising attitudes and whether increased knowledge of mental health in the broader Sri Lankan population can meaningfully reduce negative attitudes towards people with mental health problems. Additionally, considering the compounding effects of multiple crises in the country and its effects on the mental wellbeing of Sri Lankan adolescents regardless of area of residence, further exploration of the impact of these crises stratified according to area of residence may be warranted in this population.
3. Schools are a key place to promote mental health. Our findings support WHO’s mental health-promoting schools initiative, where mental health programmes integrated into the school curriculum that take an educative and behaviour-oriented approach can provide adolescents with skills to positively manage their thoughts and emotions to build positive identities and healthy relationships.
4. Considering Sri Lankan adolescents’ knowledge of informal care avenues, a more culturally resonant approach to addressing mental health needs in this group may be through enhancing existing support structures rather than introducing clinical pathways and other formal interventions. These can be promoted to adolescents through the above-mentioned school-based mental health programmes.
5. As school teachers play a central role in creating a positive school environment, a key driver of adolescent mental health outcomes, and as adolescents displayed positive attitudes towards help-seeking from teachers, educative and skill-building-oriented programmes can be implemented to provide teachers with tools for integrating mental health promotion into routine educational practice. Enhancing already existing programmes may also be a more cost-effective and sustainable solution.

## 5. Conclusions

Overall, we identified that Sri Lankan adolescents’ and school teachers’ understanding of mental health was rooted in Buddhist principles. Consistent with wider research on South Asian adolescents, our findings underscore that determinants across multiple environments surrounding an adolescent may influence their mental health. Participants in our study also identified schools as a primary driver of poor mental health in Sri Lankan adolescents. Finally, adolescents in our study indicated more knowledge of informal, rather than formal, care avenues. These findings highlight a multi-pronged approach to promoting mental health to Sri Lankan adolescents, including leveraging culturally contextualised language, diversifying mental health research, enhancing school-based mental health promotion and integrating mental health promotion into routine educational practice, as well as strengthening existing mental health promotion strategies.

## Figures and Tables

**Table 1:** Interview guide—topics and questions for interviews with 28 adolescents and 14 teachers, conducted between July and September 2023 at seven secondary schools in Gampaha District, Sri Lanka.

**Knowledge of and attitudes towards mental health** What comes to your mind when you hear the words “mental health” or “depression”? What type of behaviours do you associate with people who have good mental health? What do people who are mentally well look like? What do you think of when you hear the words ‘poor mental health’? How do you describe poor mental health? What terms have you heard other people use to describe mental health? How are people with mental health problems perceived or treated within your community/family/friends? (for adolescents only) How are students with mental health problems perceived or treated within your school? (for teachers only)
**Beliefs of causes and risk/associated factors of mental health problems and poor mental wellbeing** What do you think causes someone to have mental health problems or poor mental wellbeing? Do you think poor mental health is a problem for adolescents in Sri Lanka?
**Knowledge of care avenues for mental wellbeing (for adolescents only) ^1^** Do you know where you can go or who you can go to, to speak about mental health with? If you are having any personal, emotional, or mental wellbeing-related problems, is there anyone at your school that you can speak to about this?

^1^ Questions marked ‘for adolescents only’ were only asked to adolescent participants; questions marked ‘for teachers only’ were only asked to teachers.

**Table 2:** Demographic characteristics of 28 adolescents and 14 teachers who were interviewed between July and September 2023 at seven secondary schools in Gampaha District, Sri Lanka (N = 42).

	Sex		School Classification		
	Female	Male	Urban	Semi-Urban	Rural
**Adolescents (N = 28)**	13	15	16	4	8
**Teachers (N = 14)**	13	1	8	2	4

## Data Availability

The datasets used and/or analysed during the current study are available from the corresponding author on reasonable request due to ethical reasons.

## Supplementary Materials

The following supporting information can be downloaded at https://www.mdpi.com/article/10.3390/healthcare14030311/s1.

## Abbreviations

The following abbreviations are used in this manuscript:

LMIC	Low-and-Middle-Income Country
HIC	High-Income Country
WHO	World Health Organization
