# Supplementary material for: Sri Lankan School Student and Teacher Perspectives of Adolescent Mental Health and Its Determinants: A Qualitative Exploration
Source: Healthcare (Basel). 2026 Jan 26;14(3):311. doi: 10.3390/healthcare14030311 (PMC12896570; doi:10.3390/healthcare14030311)
Supplement: Supplementary file 1 [file healthcare-14-00311-s001.zip › healthcare-3925689-supplementary.pdf]

Supplement S1

**Code**	**Sub-Code**	Description	**Key Examples in Data**
Conceptualisation of mental health- how adolescents and teachers understand mental health			
**Knowledge of mental health**	Good mental health, poor mental health	Adolescents and teacher's description of mental health. This can include how mental health is defined by participants and also how mental health is defined/understood by the wider Sri Lankan public.<br><br>Code adolescents and teachers' description of poor and good mental health.<br><br>Code adolescents and teachers' description of specific types of mental health problems.	• Good mental health: freedom, balance, stable, confidence, happiness, laughter<br>• Poor mental health: gloomy, sad, upset, don't talk much, unhappy, don't work well with others, can't face challenges
Causes and risk factors of poor mental health outcomes			
**Causes and risk factors of poor mental health**	Individual factors, immediate environmental factors, structural factors	Adolescents and teacher's description and knowledge of causes of poor mental health.<br><br>Adolescents and teacher's description and knowledge of risk factors to developing poor mental health outcomes.	• School stress, academic pressure, relationship problems, family problems, experiencing abuse from teachers<br>• Structural factors: poverty, economic problems
Attitudes towards people with mental health-related problems			
**Stigma**	Public stigma, self-stigma	Adolescents' and teachers' description of society's discriminatory response towards people experiencing poor mental health or mental health problems<br><br>Adolescents' and teachers' internalization of public stigma	• Social stigma: crazy, junkies, vagabonds<br>• Participants indicate that community ostracizes, shuns, and stigmatizes people with mental health problems

**Positive perceptions**		Adolescents and teacher's description or/and opinions or/and beliefs of mental health or people with mental health problems in a manner other that with stigmatizing attitudes	• Adolescents try to help people that are mentally down, will treat people with mental ill health just as they would treat a person with good mental health
Knowledge of care avenues for when adolescents experience mental health-related concerns			
**Knowledge of care avenues**	Formal support sources, informal support sources	Adolescents' knowledge of the types of formal and informal mental health information sources and services available to young people in Sri Lanka.	• Knowledge of doctors, formal health services, phone services<br>• Accessed informal care such as parents, friends, teachers help, some have accessed formal care such as psychiatrists and doctors
